# Sclerostoma Tetracanthum*A paper read before the Iowa State Veterinary Medical Association.

**Published:** 1892-12

**Authors:** G. L. Buffington

**Affiliations:** Mt. Pleasant, Iowa


### SCLEROSTOMA TETRACANTHUM*.

By G. L. Buffington, D.V.M., Mt. Pleasant, Iowa.

In presenting this paper to this Association I do not wish to convey the idea that I am a specialist in this line of work or that I have made any special scientific investigation of the parasite under consideration. I simply present the subject from a clinical standpoint, as observed in my practice, referring when necessary to some of the standard authors. The literature on this subject being so meagre, the loss occasioned by the parasite so great, it behooves those who have observed its ravages to draw attention to the facts and thus elict a discussion which may prove of benefit to the profession. It is with this intention I present this paper for your consideration.

For the minute anatomy I am indebted to "Neumann's Parasites and Parasitic Diseases of Domestic Animals."

Description.—The Sclerostoma Tetracanthum is one of the very small nematodes inhabiting the intestines of the horse. The

*A paper read before the Iowa State Veterinary Medical Association.

body is slightly tapering anteriorly, of a reddish brown color when preserved in alcohol, but while in the intestinal canal of the host the larger ones are of a bright red and the smaller ones a dirty white color. They are from one-fourth to three-fourths of an inch long, the females being a little larger than the males. The mouth is circular, with a salient rim that has a crown of triangular teeth, and outwardly six papillæ, two lateral, small, and on each side of them two others, conical and very prominent. The buccal capsule is cylindrical. The caudal pouch of the male is simply excised on the ventral surface.

The posterior lines are trifurcated, the middle doubled and the anterior cleft. In the female the tail terminates in a point and the vulva is very near the anus The digestive canal is complete.

The sclerostoma tetracanthum inhabits the caecum and colon of the horse often in company with the strongylus armatus, another troublesome equine parasite and one which formed the subject of an interesting and valuable paper at the last session of the United States Veterinary Medical Association, by Dr. J. F. Winchester.

Round of Life.—The ova are segmented in the uterus. They are laid in the intestine of the host, passed out with the fæces, and if the proper conditions, warmth and moisture, are met with, will hatch out in a few days. The external phases in their development are, according to Bailliet, analogous to those of the strongylus armatus and are about as follows: If the conditions of warmth and moisture continue favorable after the ova are hatched out, they gradually grow, their integument becomes folded and forms a kind of sheath in which the worm moults. It is at this period they enter the body of the host in the water the animal drinks, or perhaps on the green forage when on low damp ground, pass into the intestines and it is probable that they encyst themselves directly into the mucous membrane of the colon and cæcum without penetrating the circulatory apparatus. At least no wandering parasites of this kind have ever been observed. They remain imbedded beneath the mucous membrane until they attain sexual maturity, when they again enter the intestinal canal to pass the remainder of their lives

Effect on the host.—As the result of the association of two organisms with more or less constancy, various modifications of form and function must occur. In some of the lower forms of animal life, where the host is but little higher than the parasite which infests it, the latter sometimes has a mechanical transforming effect on the host without seriously interfering with its function.

In the higher animal, however, the effects on the host are more serious and more markedly pathological.

Leuckart distinguishes pathological effects as due either to growth and increase of parasites, to their wanderings within the body or to the very considerable loss of nourishment which a number of parasites of appreciable size necessarily entails. The mechanical effects also are often of a serious nature. Thus parasitic worms by their size or number sometimes close up passages, as blood vessels or the bronchial tubes, often causing fatal results.

According to the old hypothesis, the pathological state of the host was not the result of the parasite, but was the cause, and in fact created them. And in spite of the gradual unravelling of the mysteries of origin and life history, medical men long clung conservatively to the old hypothesis of spontaneous generating. The ingenious experiments of Pasteur have, however, prevailed against all refractory opposition, and peremptorily established the fact that spontaneous generation could not be invoked even for the infinitely small parasites or microbes. So it is not until within the last fifty years, that with the rise of experimental helminthology, medical science shook itself free from superstition and ignorance and devoted close attention to etiology and treatment, culminating in that systematic warfare against all forms of parasitism which now occupies so important a place in Medical and Veterinary Science.

During the winter of '91-'92 my attention was for the first time directed to the serious nature of parasitism and the unwonted loss some of these small animal organisms may cause. No other one disease has caused so much loss, during the past year, among colts, in the country in which I reside as that caused by intestinal parasites, and that mostly by the sclerostoma tetracanthum, though the large round worms (acaris megalocephala) have usually been associated with them, as also the armed sclerostoma in some cases In order to convey to you some observation of the pathological effects of this parasite on the host I will cite some cases that have come under my observation. To one in particular I will refer in detail, as it was under my direct care for three months, and I had a good opportunity of observing the cause of the disease.

Case i.—In the early part of last December an eighteen-months-old filly was brought to my hospital for treatment. The filly presented an extremely emaciated condition as though suffering from some exhausting disease. The hair was long, rough and staring; left side of the back covered with scabs, legs swollen, more especially at hocks, which presented great baggy enlargements, mucous membranes pallid, pulse about normal and temperature a little below normal. There was a constant rumbling of bowels. Not being satisfied as to the cause of the extreme emaciation, she was placed in a box stall to note any further symptoms which might be presented. It was soon observed a mild form of diarrhœa was present and the fæces were very fœtid. The appetite was ravenous and she would drink about as much water as two ordinary work horses at hard work. When down, was unable to rise without assistance though she would struggle to do so. When assisted to her feet would stand around quietly and eat most of the time. Appeared to suffer none, but was just weak and lifeless. On examing the fæces, a number of bright red worms from three to five-eighths of an inch long were found on the surface of them. Every passage contained them and they were always found on the surface, never within the contents.

The filly continued to grow weaker for four or five days, when on attempting to raise her one evening, we were unable to do so on account of her perfectly helpless condition. After lying all night and struggling most of the time she could not even raise the head from the ground on the following morning, and her case seemed almost hopeless. However, with the assistance of a sling and two or three men we finally succeeded in getting her up, and after being supported for a short time was able to stand without much assistance. She seemed to do well enough when kept on her feet, but when allowed to lie down would lie quietly for only an hour or two and then begin to struggle to rise, often injuring herself in the struggles. For this reason she was kept up by use of the sling most of the time. To expel the worms, which were now regarded as the source of all her trouble, finely pulverized iron sulph. was used for a few days in the ground feed and this followed by an aloetic purge. After purgation began the watery fæces were literally filled with these small worms, most of them of a dirty white color, with some, the largest, a bright red, and various gradation between these two. On close examination, while still warm, they looked to be a moving, writhing mass of living organism ; there being thousands of them in a small quantity collected on a shovel ; and as they continued to be expelled in this manner for thirty-six hours, it would be impossible to even make an estimate of the vast number that existed in the intestines of this filly. The greater number of these worms were about one-fourth of an inch in length. The bright red ones, which seemed to be the same kind more fully developed, would average about one-half inch in length.

Two or three weeks after the first aloetic purge another was given with about the same results, with the addition of expelling a few of the large round worms. The filly improved very slowly and could not rise without assistance for two months.

During all the time this case was under my observation her appetite was ravenous, thirst great, coat rough and staring and emaciation extreme, improving, however, a little during the last three or four weeks. Diarrhœa was present most of the time and the fæces were very fœtid. Occasionally the bowels would be normal for a few days and then diarrhœa would supervene, this condition gradually improving until the bowels finally became normal. She continued all the time to pass those red worms on the surface of the fæces. This animal received, besides the vermifuges, vegetable tonics and an abundance of good nutritious food. The last month powd. nux vomica only was used, given in the feed. At the end of three months she was taken home, well fed, including a bucket of milk every day which she greedily drank, and after being turned to pasture in the spring soon fattened up and did remarkably well, and now at two and a half years of age weighs nearly twelve hundred pounds. I might add that during the last few weeks, since the beginning of cold weather, she shows symptoms of springhalt. Two or three other colts on the same farm were affected with the same kind of worms, but not so badly.

Case II.—During the early part of last January was called to see a herd of twelve colts, consisting of yearling and two-year-olds. They had not been thriving the previous Summer and Fall, and had been gradually losing flesh, but no particular attention was paid to them until at this time, when they began to be so reduced as to be unable to stand. One had died four or five weeks previous to my visit. Three were down, unable to rise, and pitiable looking objects indeed. Five or six more were greatly emaciated, but could still get around. The remaining two or three appeared lively and well, but were also much reduced in flesh. The three that were down presented about the same symptoms as case No. I. The worst one died a few days later, and at the post mortem, several days after death, I found the whole mucous surface of the colon and cæcum was literally lined with these small worms, sclerostoma tetracanthum. They were very loosely attached by anterior extremity to the mucous membrane. The attachment was, however, very loose, as they could easily be brushed off. I did not find any encysted beneath or within the mucous membrane. They had probably, previous to death of host, all become sexually mature, and passed into the intestinal canal. A portion of the small intestines, five or six feet in length, was filled with the large, round worms (ascaris megalocephala). There were two or three hundred of them, and were all matted together, completely filling the canal. They measured from four to twelve inches in length. This colt, though a small one, had, besides harboring all these worms, been infested with great numbers of lice previous to death. The other two which were found unable to rise were helped up every morning and well cared for, but, after lingering along several weeks, finally died. The post mortem revealed about the same condition as the first one, except there were only a few of the large, round worms, and in addition a number of white worms, from one-half to two inches long, were found imbedded beneath the parietal layer of the peritoneum. These proved to be the strongylus armatus. These might have existed in the first one and escaped my notice, as I did not look especially for them. Another one of the colts, being very weak, in some manner fell and rolled down an embankment, from the effects of which it died. Post mortem revealed about the same condition as last two. Also found portion of the colon filled with blood clot, probably the result of the fall. The remainder of the herd, with good care, succeeded in getting through the Winter, and began to gain as soon as turned to grass. They all received, as medicinal treatment, iron sulph., followed by an aloetic purge, and then the iron sulph. combined with vegetable tonics.

Case III.—My attention was called to another herd, consisting of ten weanling and one or two yearling colts, which were not thriving, although they received the best of care, being well bred, some of them standard bred. On examining fæces, found they contained the small, red worms. They showed no symptoms of diarrhœa, and were not yet extremely emaciated. They were also affected with lice. The worms were eliminated by the same treatment given the others, and they all recovered. There were many older horses on the same farm, all of which remained free from the parasites.

Numerous other cases of a similar nature were reported from various localities within the county. Although I did not see them all, I was led to believe they were from the same cause.

Dr. G. W. Butler, of Ohio, in Journal of Comparative Medicine and Veterinary Archives, Vol. XI, No. 9, also in

*American Veterinary Review.* Vol. XIV, No. 6, refers to this parasite (sclerostoma tetracanthum) occurring as a coincidence in some horses affected with rabies. On post mortem, he found a great number imbedded in the mucous membrane of the colon and cæcum, as well as a large number free in the intestinal canal. He also, in the same article, quotes a letter received of Dr. T. S. Butler, then of Iowa, in which he refers to the same parasite as existing in a number of horses, one of which, a four-year-old mare, died from their effects, and on post mortem he also found a vast number infesting the mucous membrane, and a careful estimate indicated that there were not less than seventy-five thousand of these parasites infesting the large intestine of this animal, and this notwithstanding that the animal had passed in the fæces, during the two weeks preceding death, an equally large number. In the intestines were also found many of the large, white worms mentioned by Prof. Williams, and measuring from one to two inches in length. Williams also mentions this worm as existing in the mucous membrane In the same subjects he found others in the intestinal canal, which were from two to three inches long, and he was of the opinion that these were of a different variety. There were none of the white worms found in the intestines of any of those cases examined by me, and, as stated before, I found no worms of any kind encysted in the mucous membrane of the colon or cæcum.

From some experiments performed by Dr. T. S. Butler with these small, red worms, mentioned in the letter referred to above, he succeeded in growing them to double their original length, during which time they lost the red color and became much like the white worms which he found. As the result of this, he was led to believe that probably the white worm was only an advanced stage of the small, red worm.

Colts seem to be more susceptible to the attacks of these worms than older horses. This, perhaps, is due to the fact that the intestinal walls are more delicate and consequently offer less resistance to the burrowing of the parasite in the first stage of its development. In only two instances have I observed these worms in adult horses in any great numbers. In these two cases there was no emaciation ; the only symptoms being those of acute diarrhœawhich soon terminated in recovery.

Prevention.—Though we may not yet know the precise life history and mode of introduction of these parasites, yet we are not altogether at the mercy of their invasion. The ova, it is pretty certainly determined are derived from without, in all probabilities from the water supply or from forage when obtained on low, damp ground. It is evident, then, if a vigorous quarantine can be kept over these supplies, we can expect to keep animals reasonably free from invasion. But as this requires continual patient care, and on many farms difficult to control, it is generally neglected in practice, and the necessity for it comes into notice only after losses have been endured.

The parasite does not always come, however, as the result of faulty hygienic surroundings, as animals may be infested when running in good pasture and drinking from a stream of running water. And yet, after one attack under such circumstances, it would be advisable to remove the animals to a higher or better-drained pasture, and furnish the water supply from a deep well which receives no surface drainage. Iron sulph. mixed with the salt, and given once or twice a week, would prove beneficial in warding off the parasite. If an attack is imminent, the iron should be given more frequently, mixed with ground feed.

Treatment.—The indications in the treatment of this form of parasitism is to cause expulsion of the parasite and maintain the strength of the patient by tonics or stimulants, if the animal is greatly debilitated.

Various and numerous are the therapeutical agents used as vermifuges. Arsenious acid, tartar emetic, santonine, male shield fern, empyreumatic oil, have all been used with more or less success. Turpentine is highly lauded by some authorities as an efficient agent in expelling these worms. The treatment proving most satisfactory to me has been iron sulph., given for two or three days in ground feed, and this followed by a dose of aloes sufficient to cause mild purgation, the dose of course depending on the age and condition of the animal; this again followed by the iron and vegetable tonics, where debility is great. Good, nutritious food in abundance and pure water are essential, as is also shelter from the extreme cold. After an animal becomes so much emaciated as to be unable to stand, only the most careful nursing will insure recovery.

The geographical distribution of this parasite is not yet known, as so little has been written upon the subject. I am informed by Dr. C. W. Stiles, of the Bureau of Animal Industry, after sending him some specimens of the worms found in Case I, which he pronounced as given above (sclerostoma tetracanthum), that the Bureau has had quite a number of cases of them reported from Minnesota and Illinois. To what extent they exist in Iowa I shall hope to determine from the discussion of this paper.

